# Surgical Bailout of Transcatheter Aortic Valve Embolization Using a Right Anterior Minithoracotomy Approach

**DOI:** 10.1177/15569845241248657

**Published:** 2024-05-09

**Authors:** Anthony D. Sinobas, Bleri Celmeta, Arturo Bisogno, Tommaso Viva, Antonio Miceli, Vito Domenico Bruno, Mattia Glauber

**Affiliations:** 1 University of Bristol Medical School, UK; 2 Minimally Invasive Cardiac Surgery Unit, IRCCS Ospedale Galeazzi – Sant’Ambrogio, Milan, Italy; 3 Department of Cardiac Surgery, Vita-Salute San Raffaele University, IRCCS San Raffaele Scientific Institute, Milan, Italy

## Introduction

Aortic stenosis (AS) is a relatively common valvular disease in elderly patients. Current guidelines highlight evidence for treatment of severe AS using surgical aortic valve replacement (SAVR) and transcatheter aortic valve implantation (TAVI).^1^ Opting for TAVI enables patients deemed higher risk for surgery to be treated effectively. As with all interventions, TAVI is not without risks; surgical bailouts are performed in 1% of cases and associated with poor survival rates at approximately 50%.^2^

We present a patient who experienced bioprosthesis migration into the ascending aorta after TAVI, requiring urgent surgical explantation via a right anterior minithoracotomy (RAMT) and subsequent SAVR. The step-by-step procedure is described in the Supplemental Video.

## Case Report

We present the case of an 83-year-old former smoker with a history of chronic obstructive pulmonary disease, hypertension, and dyslipidemia on statin treatment. They were referred in May 2023 for severe AS with maximum and mean pressure gradients of 90 mm Hg and 40 mm Hg, respectively; a EuroSCORE II of 2.16%; Society of Thoracic Surgeons score of 5.52%; and a KATZ frailty index of 6 (independent patient). TAVI was proposed after the case was discussed in our Heart Team, noting the patient’s preferences and older age. Fully informed written consent was obtained directly from the patient prior to the operation.

**Fig. 1.**
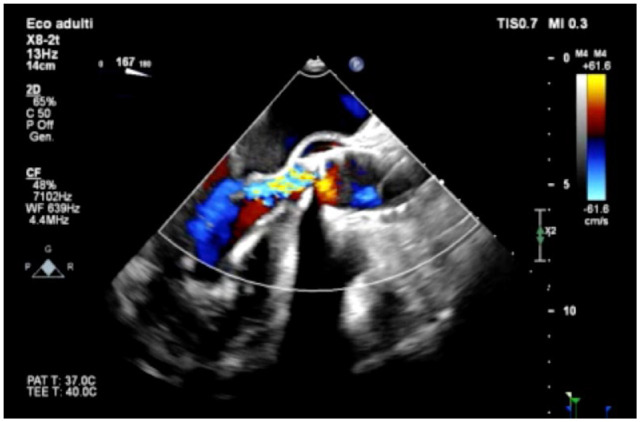
Post–transcatheter aortic valve implantation procedure transesophageal echocardiography showing significant aortic regurgitation due to the embolized bioprosthesis.

In September 2023, the patient underwent a percutaneous transcatheter Hydra 30 mm bioprosthesis valve (Vascular Innovations Co Ltd, Nonthaburi, Thailand) implantation via the right femoral artery. Immediate postprocedural prosthesis migration into the sinotubular junction was noted, causing severe stenosis with an index aortic valve area of 0.4 cm^2^/m^2^ and moderate regurgitation (Fig. 1). The patient showed hemodynamic stability with no pericardial effusion and was taken to the intensive care unit (ICU). Serial echocardiographic scans demonstrated a slight progression of the migration of the bioprosthesis. After careful review using 3-dimensional computed tomography (CT), echocardiography scans, and accounting for the patient’s wishes, we decided to perform an urgent surgical intervention. The preoperative CT scan demonstrated a relatively large second intercostal space, with the ascending aorta course tendentially on the right of the sternum. A minimally invasive approach through the second intercostal space was deemed optimal for easy and direct access to the migrated prosthesis. We proceeded with an urgent prosthesis explantation and surgical aortic sutureless bioprosthesis implantation (Perceval Plus, size L; Corcym, London, UK) using a RAMT approach. After an ICU stay of 3 days, the patient experienced a single episode of atrial fibrillation with rapid ventricular response, successfully cardioverted using amiodarone. Right thoracocentesis was also necessary. Symptoms of dysuria were described, prompting empirical antibiotic treatment. After a total hospital stay of 10 days, the patient was transferred to another center to undertake a cardiac rehabilitation program.

### Surgical Procedure

A 6 cm incision was made anteriorly in the right second intercostal space. Access to the mammary pedicle was gained, with interruption achieved using LIGACLIP (Ethicon, Raritan, NJ, USA) and scissors. A soft-tissue retractor was placed, and the pericardial sac was opened, ensuring adequate clearance from the phrenic nerve with a margin of ≥4 cm. Pericardial stay sutures were placed, enhancing visualization. The left femoral artery was exposed, and a Bio-Medicus 19F cannula (Medtronic, Dublin, Ireland) was introduced. After echocardiography-guided percutaneous bicaval cannulation via the right femoral vein with a 25F sized cannula, cardiopulmonary bypass (CPB) was initiated. The cardioplegia cannula was positioned in the ascending aorta, and the detachable-branch Glauber clamp was used for aortic cross-clamping.

**Fig. 2.**
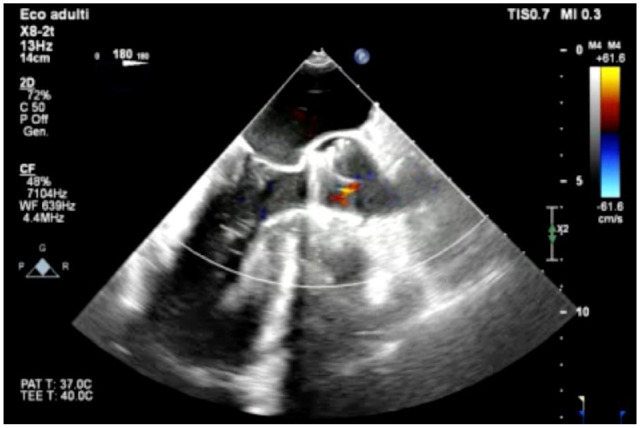
Post–surgical aortic valve replacement transesophageal echocardiography showing absence of intravalvular and paravalvular regurgitation.

Del Nido solution was administered in the aortic root under transesophageal echocardiography (TEE) surveillance for the valve continence and left ventricle inspection. The antegrade cardioplegia successfully induced full cardioplegic arrest. The aortotomy was subsequently performed, higher than usual due to the malposition of the migrated prosthesis. After visualizing the prosthesis, 2 pairs of Kelly hemostat forceps were used, clasping the circular wire mesh. Simultaneously twisting and lifting both forceps allowed the prosthesis to be explanted without trauma or damage to the aorta. The calcified native aortic cusps were removed. Three polypropylene sutures were placed at the nadir of the 3 cusps, guiding implantation of a sutureless bioprosthesis. The valve was expanded to its final size, and the insertion tool was detached. The aortotomy was closed in a standard fashion, deairing maneuvers were performed, and the aorta was unclamped. Valve competence was checked using intraoperative TEE, and no intravalvular or paravalvular leak was present (Fig. 2). After CPB weaning, femoral and arterial cannulas were removed and purse strings tied. The heart was checked for adequate hemostasis, and the pericardial sac was partially closed using polypropylene continuous sutures. The thoracotomy and the groin were closed in a standard fashion.

## Discussion

Currently, TAVI is recommended in patients deemed at high risk for surgery (EuroSCORE II >8%) and elderly (≥75 years old), while SAVR is reserved for those who are younger and at lower surgical risk.^1^ However, the best treatment should be tailored on an individual basis by experts constituting the Heart Team.

Despite advancements in TAVI, intraprocedural and postprocedural risk remains evident. The TRAVEL Registry documented that of 29,636 patients, 273 patients undergoing TAVI experienced valve embolization and migration (TVEM), with 30-day and 1-year mortality shown as 18.6% and 30.5%, respectively. Of the 273 TVEM cases, repositioning attempts were made in 112 patients and successful in 51.8%, whereas 19% required conversion to surgery.^3^

Cardiac surgery has traditionally maintained a central role in the management of complications following TAVI. Current guidelines in Europe and the United States recommend TAVI to be performed in “Heart Valve Centers,” with access to a multidisciplinary team consisting of interventional cardiology, cardiac anesthetists, imaging specialists, and cardiac surgeons available onsite.^1,4^ Traditional full sternotomy surgical management of TAVI complications has been assessed in various studies.^5,6^

Salem et al. highlighted in 2,048 patients who underwent TAVI that just over 1% of patients required SAVR.^5^ Despite the relatively small fraction experiencing complications requiring surgery, the study also noted poorer outcomes in this group and a survival rate of about 50%. Similar rates were reported by Li et al., with 3.88% of patients undergoing emergency cardiac surgery, 65% survival at 1 year, and just over 50% survival at 5 years.^6^

While studies describe urgent surgery after TAVI in a full sternotomy approach, to our knowledge no cases of a minimally invasive approach have been reported in this context. First described by Benetti et al., the use of RAMT has revolutionized the practice of aortic surgery using a sternal-sparing approach.^7^ Bakhtiary et al. investigated patients undergoing isolated aortic valve surgery, demonstrating that, even among minimally invasive approaches, RAMT optimizes outcomes reducing aortic cross-clamp times, time to mobilization, and reoperation rates when compared with a partial sternotomy.^8^

At 83 years of age with a migrated percutaneously implanted aortic valve bioprosthesis, the patient was successfully operated in an urgent setting by RAMT approach. Careful preoperative surgical planning is mandatory; the thoracic CT scan demonstrated feasibility of the procedure, assessing the anatomical position of the aorta. Antegrade cardioplegia was sufficient to safely induce cardioplegic arrest; otherwise, selective antegrade cardioplegia would have been necessary after bioprosthesis explantation. A sutureless valve was chosen to facilitate implantation and reduce cross-clamp time. Differently from percutaneously implanted valves, sutureless bioprostheses are implanted under surgical direct vision and benefit from an extensive decalcification and complete native valve removal.

In conclusion, we believe that when performed in high-volume minimally invasive cardiac surgery centers, sternal-sparing surgical approaches provide benefit in older, frailer, higher-risk patients, even in an urgent context.

## Supplemental Material

sj-jpg-1-inv-10.1177_15569845241248657 – Supplemental material for Surgical Bailout of Transcatheter Aortic Valve Embolization Using a Right Anterior Minithoracotomy Approach Supplemental material, sj-jpg-1-inv-10.1177_15569845241248657 for Surgical Bailout of Transcatheter Aortic Valve Embolization Using a Right Anterior Minithoracotomy Approach by Anthony D. Sinobas, Bleri Celmeta, Arturo Bisogno, Tommaso Viva, Antonio Miceli, Vito Domenico Bruno and Mattia Glauber in Innovations

sj-tif-1-inv-10.1177_15569845241248657 – Supplemental material for Surgical Bailout of Transcatheter Aortic Valve Embolization Using a Right Anterior Minithoracotomy Approach Supplemental material, sj-tif-1-inv-10.1177_15569845241248657 for Surgical Bailout of Transcatheter Aortic Valve Embolization Using a Right Anterior Minithoracotomy Approach by Anthony D. Sinobas, Bleri Celmeta, Arturo Bisogno, Tommaso Viva, Antonio Miceli, Vito Domenico Bruno and Mattia Glauber in Innovations
