# Supplementary figures and images for: Surgical Bailout of Transcatheter Aortic Valve Embolization Using a Right Anterior Minithoracotomy Approach
Source: Innovations (Phila). 2024 May 9;19(3):327–9. doi: 10.1177/15569845241248657 (PMC11393425; doi:10.1177/15569845241248657)

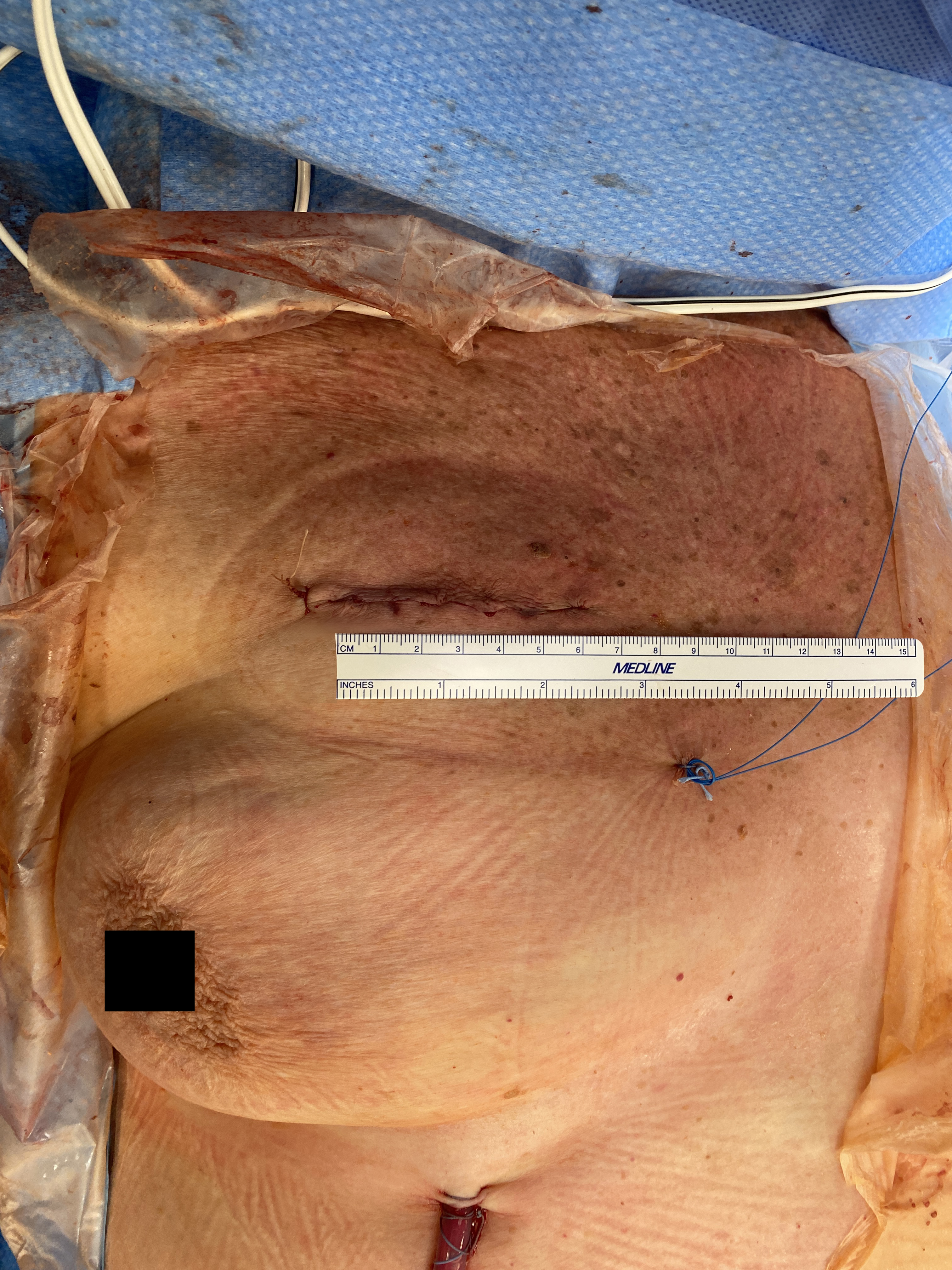

Supplement: sj-jpg-1-inv-10.1177_15569845241248657 – Supplemental material for Surgical Bailout of Transcatheter Aortic Valve Embolization Using a Right Anterior Minithoracotomy Approach [file sj-jpg-1-inv-10.1177_15569845241248657.jpg]

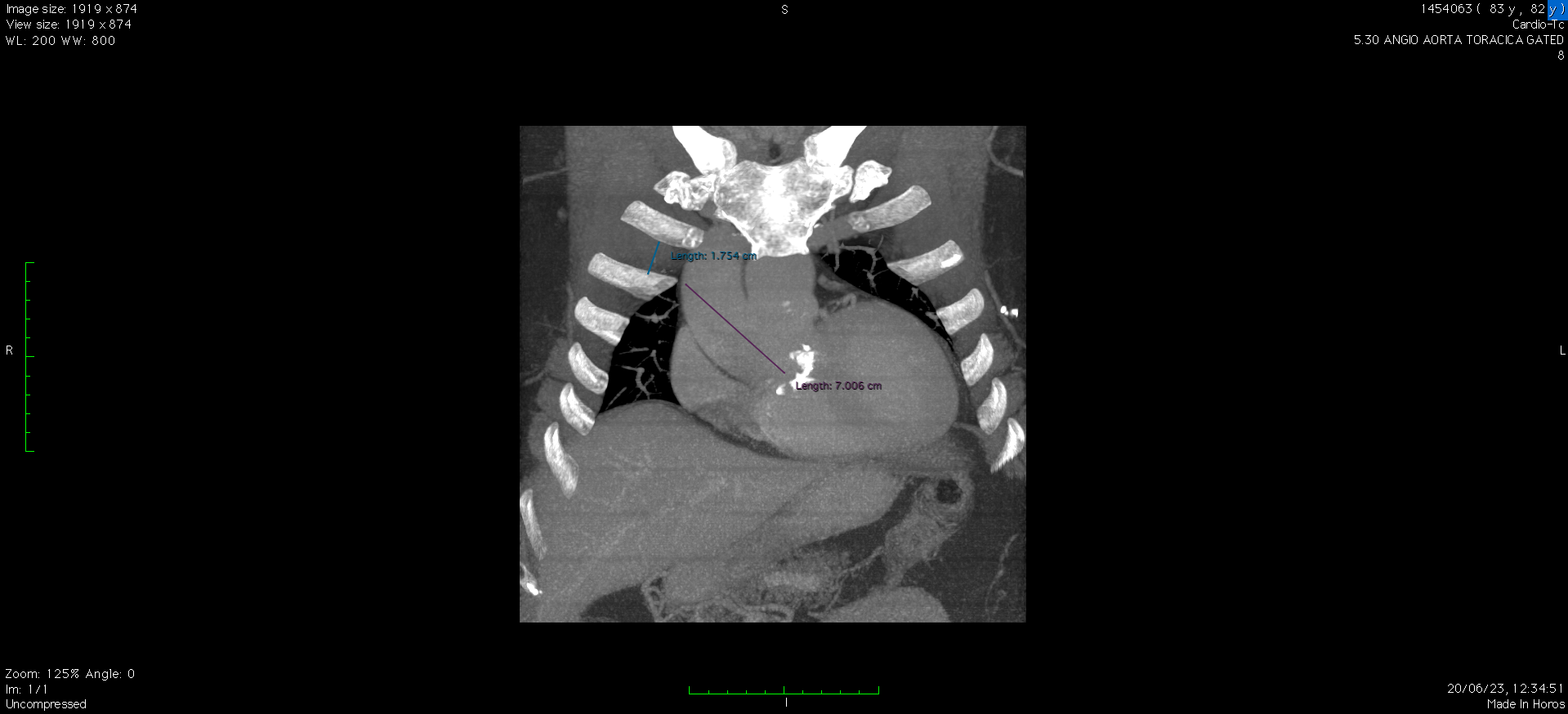

Supplement: sj-tif-1-inv-10.1177_15569845241248657 – Supplemental material for Surgical Bailout of Transcatheter Aortic Valve Embolization Using a Right Anterior Minithoracotomy Approach [file sj-tif-1-inv-10.1177_15569845241248657.tif]
